# Genome Sequence of “*Candidatus* Thioglobus singularis” Strain PS1, a Mixotroph from the SUP05 Clade of Marine Gammaproteobacteria

**DOI:** 10.1128/genomeA.01155-15

**Published:** 2015-10-22

**Authors:** Katharine T. Marshall, Robert M. Morris

**Affiliations:** School of Oceanography, University of Washington, Seattle, Washington, USA

## Abstract

Mixotrophic marine bacteria from the SUP05 clade are ubiquitous in the ocean. Here, we announce the complete genome sequence of “*Candidatus* Thioglobus singularis” strain PS1, the first cultured mixotrophic representative from the SUP05 clade.

## GENOME ANNOUNCEMENT

Mixotrophic members of the SUP05 clade of marine gammaproteobacteria are cosmopolitan and have important roles in marine biogeochemical cycles (1–5). They are of particular interest because of their potential to oxidize sulfur and fix carbon in the dark ocean and in expanding low oxygen zones (3, 5). Here we announce the complete genome sequence of “*Candidatus* Thioglobus singularis” strain PS1, which was isolated from surface waters (5 m) in Puget Sound. “*Ca.* Thioglobus singularis” PS1 is a member of the *arctic96BD-19* subclade (designated here by lower case italics) and is the first cultured representative from the SUP05 clade (6). It has a circular chromosome that is 1,714,148 bp in length and codes for 1,750 genes.

Cultures of “*Ca.* Thioglobus singularis” were grown in 1-liter polycarbonate bottles of filter sterilized seawater media as previously described (6). Cells were then filtered onto sterile 0.2-µm polyethersulfone filters (Pall, Port Washington, NY), placed in 15-mL Teflon tubes containing 2 mL of sucrose lysis buffer (SLB), and flash frozen in liquid nitrogen. Cells were later lysed by adding 100 µL of 1 mg/mL lysozyme and incubating at 4°C for 60 min, then by adding 465 µL of 10% SDS and 250 µL of proteinase K and incubating at 55°C for 2 h. DNA was extracted and purified from cell lysates using DNeasy blood and tissue and MinElute kits according the manufacturer’s instructions, respectively (QIAGEN, Germantown, MD). A total of 20.35 µg of DNA was used to construct a mate-pair library according to the SOLiD v3.0 mate-pair protocol (Life Technologies, Foster City, CA). The “*Ca.* Thioglobus singularis” genome was assembled using SEAStAR v0.4.17 as previously described (7). The initial assembly was 97% complete and contained 177 gaps with a mean gap length of 198 bp. Gaps were closed by PCR with custom primers designed using Geneious v7.0.4 (Biomatters, Auckland, NZ). Amplification products were visualized, gel purified, and sequenced by Genewiz (Foster City, CA). The complete genome sequence (1,714,148 bp) was confirmed by performing additional PCRs to resolve any irregularities in the assembly and by comparing read coverage, physical coverage, and insert sizes of mate pairs covering the genome. Annotations were performed using the NCBI Prokaryotic Genome Automatic Annotation Pipeline and were checked against RAST annotations (8, 9), IMG annotations (10), and in some cases by phylogenetic analyses. Discrepancies were corrected and final annotations were submitted to NCBI.

### Nucleotide sequence accession number.

The complete genome sequence of “*Ca.* Thioglobus singularis” strain PS1 is available in GenBank under accession number CP006911.
